# Unusual case of papillary thyroid carcinoma mimicking paraganglioma in a 16-year-old girl

**DOI:** 10.1016/j.ijscr.2019.10.032

**Published:** 2019-10-23

**Authors:** Saima Ahmad

**Affiliations:** Pakistan Institute of Neurosciences, Neurointerventional Department, Lahore General Hospital, Ferozpur Road, Lahore, Pakistan

**Keywords:** AJCC, American Joint Committee on Cancer, CT, computed tomography, DSA, digital subtraction angiogram, HTT, hyalinizing trabecular tumor, IJV, internal juglar vein, MTC, medullary thyroid cancer, Paraganglioma, Immunohistochemistry, Thyroid, Papillary carcinoma

## Abstract

**Background:**

Papillary thyroid carcinoma is a common neoplasm arising from follicular cells of the thyroid. Differentiating the neck mass between thyroid paraganglioma and papillary thyroid carcinoma is often difficult.

**Case report:**

We report a 16-year-old girl with painless neck mass. Physiological goiter was suspected initially but we found papillary thyroid carcinoma.

**Results:**

CT neck revealed hyper vascular lesions in bilateral lobes of thyroid. DSA imaging was done for further evaluation which showed high flow fistulous type of lesion supplying from superior thyroidal artery raising suspicion of paraganglioma. Postoperative biopsy ultimately revealed papillary carcinoma of thyroid in the background of hashimotos’ thyroiditis with metastatic deposits in neck lymph nodes.

**Conclusion:**

Thyroid paraganglioma is a rare type of tumor, it arises from inferior laryngeal paraganglioma within thyroid capsule and is as thus difficult to distinguish from thyroid carcinomas. A biopsy is the best diagnostic choice but immunohistochemistry is the only investigation which is considered as the gold standard for distinguishing between these two diseases.

## Highlights

• Papillary thyroid carcinoma is the most widely recognized pediatric carcinoma.
• Thyroid paraganglioma is rare, arises from inferior laryngeal paraganglioma.
• It’s hard to separate from thyroid carcinomas as it arises from thyroid capsule.
• The only way to differentiate the two diseases is through immunohistochemistry.

## 1 Introduction

This paper has been written in line with SCARE criterion [1]. Papillary thyroid carcinoma is the most widely recognized pediatric thyroid carcinoma emerging from the follicular cells of thyroid as a reason in to 85–95% of cases [2]. In teenagers and young grownups, lymph node metastasis, distant metastasis and tumor progression are more often seen in younger age groups compared to older patients [3].

Paragangliomas are unprecedented neuroendocrine tumors, emerging from the neural crest cells originated paraganglia of the autonomic nervous system. Paraganglioma adjoining or inside the thyroid gland is a subset of laryngeal Paragangliomas, which was first depicted in the right upper lobe of the thyroid gland by Van Miert in 1964 [4].

Differentiating the neck mass between thyroid Paraganglioma and papillary thyroid carcinoma is occasionally difficult. Concurrent introduction has sometimes been depicted in studies. The determination of paraganglioma mostly relies upon the mixture of morphology and immunohistochemistry However, in past reports, it was made by morphology, special staining, and electron microscopy, which couldn’t recognize paraganglioma and thyroid carcinoma. Since the head and neck paraganglioma is a nonfunctional tumor, there is no clear clinical presentation other than a cervical mass. Thyroid paraganglioma, medullary thyroid carcinoma, and hyalinizing trabecular tumor (HTT) are hard to recognize just by histological morphology, so they are typically misdiagnosed before immunohistochemistry. Therefore, all thyroid paragangliomas were distinguished as thyroid carcinoma [5].

This report describes an usual case of papillary thyroid carcinoma in a young girl that was mimicked thyroid paraganglioma on imaging and digital subtraction angiography but was later confirmed on immunohistochemistry.

## 2 Case report

**Fig. 1:**
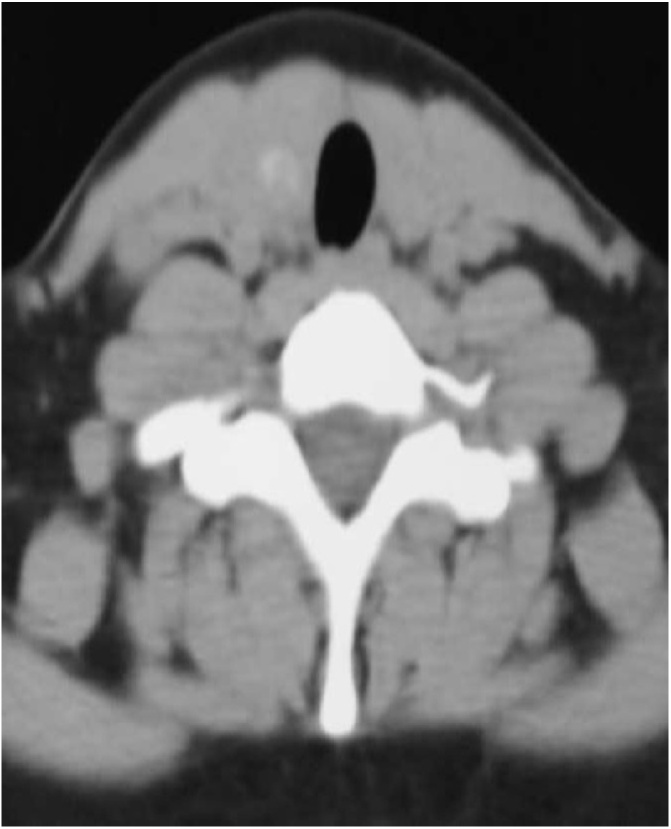
A 16 year old girl with bilateral papillary thyroid carcinoma. (A) Axial enhanced CT neck showing enhancing mass lesion involving right thyroid lobe. The left lobe appears to be normal in CT scan.

**Fig. 2:**
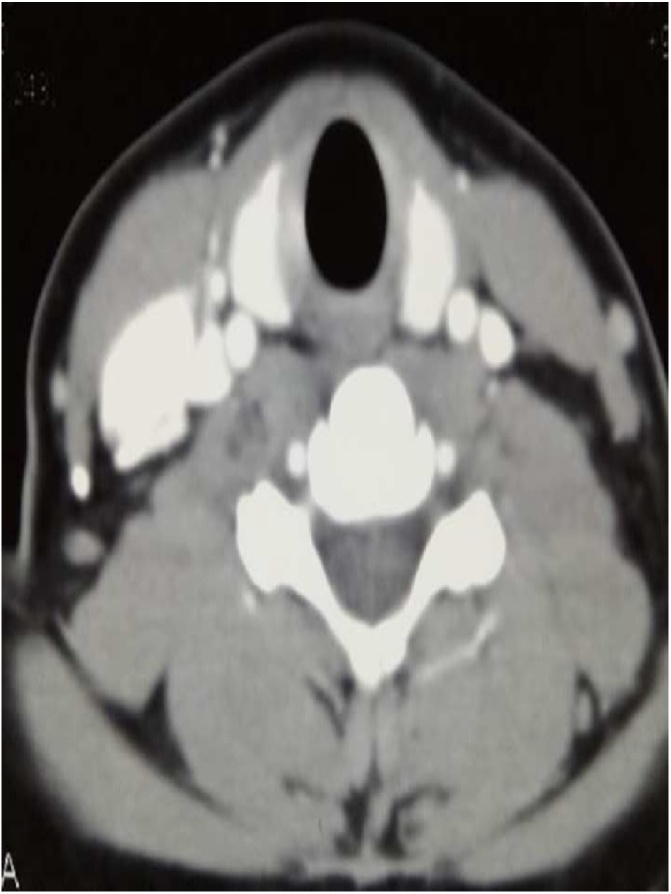
Axial enhanced CT neck showing abnormal enhanced channels on right side appear to be vascular in nature.

**Fig. 3:**
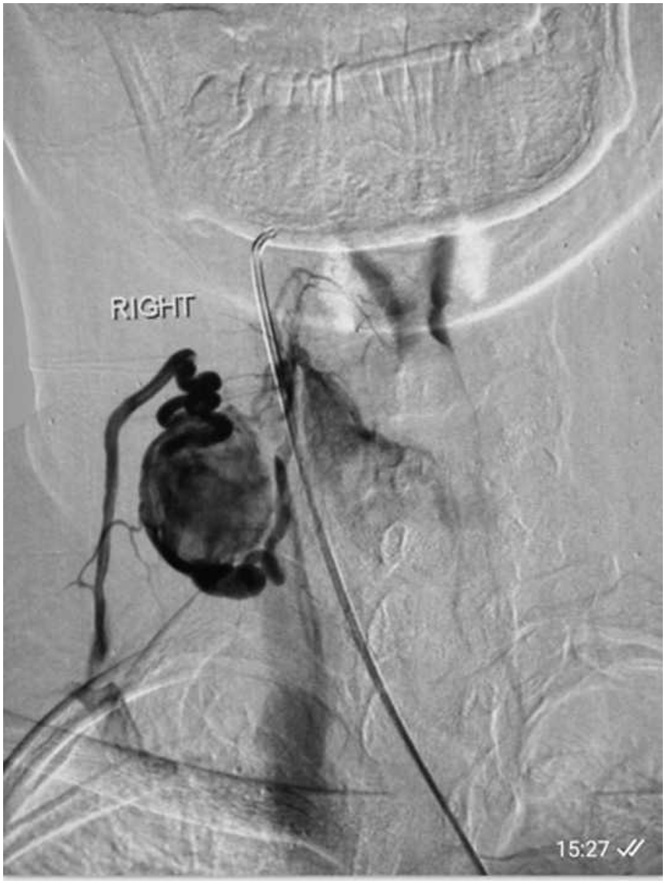
Selective right external carotid angiogram showing highly vascular fistulous type of lesion fed by superior thyroidal artery and draining into internal jugular vein corresponding to vascular lesion visible on CT neck giving rise the suspicion of thyroid paraganglioma.

A 16-year-old girl presented at our center with a six months history of multiple swellings on lateral aspect of neck on both sides. Swellings were insidious in onset and there was a gradually progressed in size. There was no history of thyroid cancer nor any history of irradiation. On examination, the thyroid gland was palpable and firm with right sided cervical lymphadenopathy. Anti-thyroglobulin antibodies and thyroglobulin levels were within normal limits. CT neck showed an enhancing mass lesion in right thyroid lobe with abnormally dilated channels (Fig. 1, Fig. 2). A digital subtraction angiogram was done due to these suspicious abnormally dilated channels. Selective angiogram of right external carotid artery revealed high flow fistulous type of lesion fed by superior thyroidal artery, draining into internal jugular vein (Fig. 3).

**Fig. 4:**
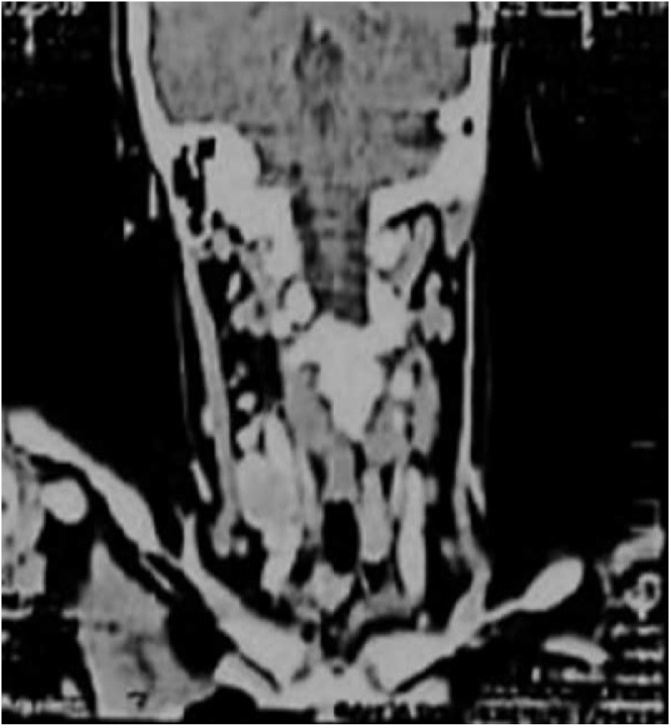
Post right thyroidectomy and isthmectomy CT neck coronal enhanced view showing metastatic nodes on right side at level IV and level VI compressing the internal jugular vein.

**Fig. 5:**
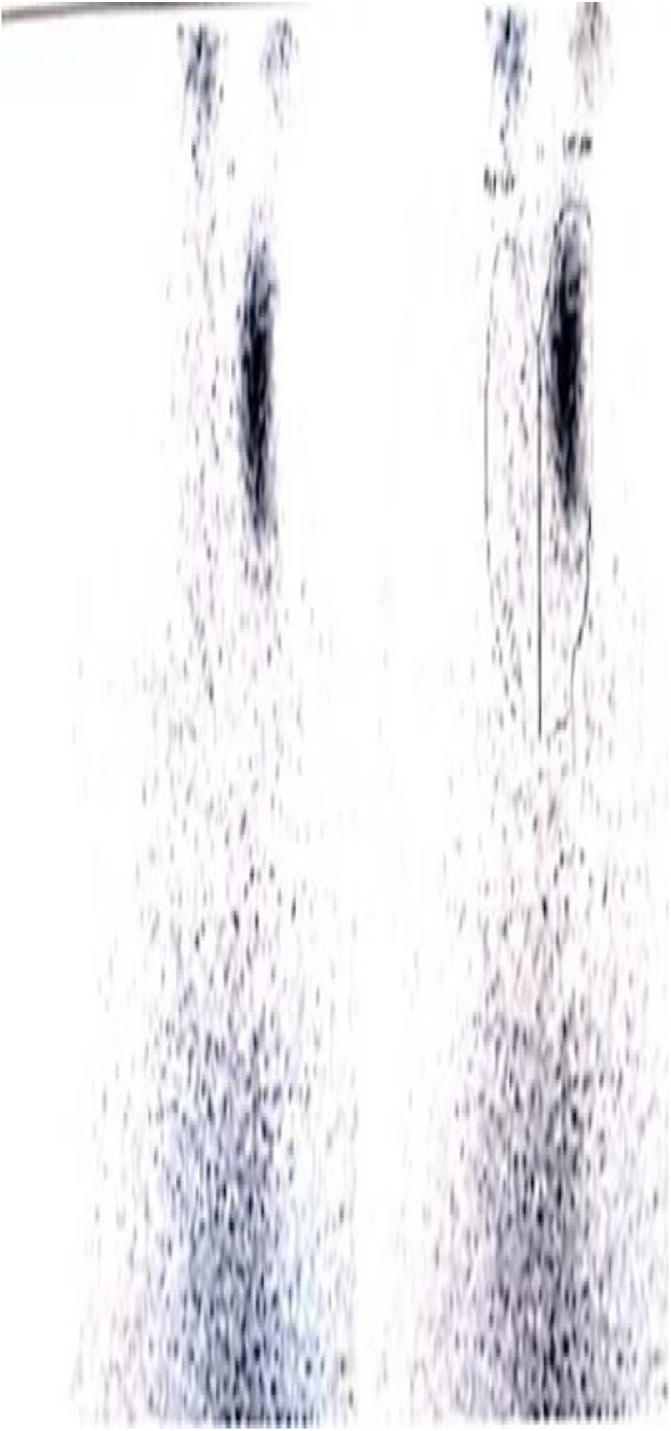
Post-operative right thyroidectomy and isthmectomy thyroid scan showing radiotracer uptake in left lobe of thyroid indicating residual thyroid papillary carcinoma.

This led to a high suspicion of thyroid paraganglioma. Right thyroid lobectomy and isthmectomy was done. Grossly the resected thyroid specimen measured 30 mm × 20 mm × 10 mm and weight was 8 g. The tumor was firm in consistency, tan white and infiltrating type. Extra thyroidal extension was not seen. Histological diagnosis was papillary carcinoma of thyroid. Post operatively thyroid scan (Fig. 5) was repeated to ensure no residual tumor. Thyroid scan showed no radiotracer uptake in the region of right lobe and isthmus, however, uptake was seen in the region of left lobe. The functioning tissue represents left lobe of thyroid. Follow up CT neck (Fig. 4) showed no mass or nodule in left lobe of thyroid. Two discrete enlarged enhancing lymph nodes were present at cervical level IV on right side measuring 24 × 14 mm and another in midline measuring 14 × 10 mm at level VI. Level IV node was compressing the internal jugular vein with a tuft of tortuous venous collaterals draining into internal jugular vein. Again suspicion of paraganglioma was raised. A second surgery was planned and left thyroid lobectomy and modified neck dissection was done on right side. Grossly the resected specimen measured 40 mm × 25 mm × 20 mm. The tumor involved whole thyroid lobe, firm in consistency and tan white in color. Microscopically showed thyroid parenchyma exhibiting a tumor arranged in papillary configuration. The papillae were lined by cells that showed crowded nuclei with overlapping of adjacent nuclei, pale to clear chromatin, irregular nuclear contours and nuclear grooves. Rare intra nuclear cytoplasmic inclusions were also found. Histological diagnosis was residual foci of papillary carcinoma arising in the background of Hashimoto’s thyroiditis. 2 out of 8 lymph nodes were positive for metastatic carcinoma, the largest deposit measured 10 mm. There was no lymphatic invasion. Extra nodal extension was not seen. Pathological stage classification was (pT1a) – as per the AJCC 8th Edition. Suspicion of thyroid paraganglioma along with papillary carcinoma was still there so further immunohistochemistry was done.

IHC analysis was negative for chromogranin, synaptophysin and S-100 and positive for calcitonin which further confirmed papillary carcinoma and absence of thyroid paraganglioma.

## 3 Discussion

Thyroid paraganglioma are uncommon tumors that emerges from the inferior laryngeal paraganglioma [4]. The inferior laryngeal paraganglioma can be situated inside thyroid capsule clarifying why this tumor may happen in thyroid gland [6]. Most patients are female and present with an asymptomatic thyroid mass. It is a diagnostic trap and once in a while misdiagnosed as follicular neoplasm [4].

Literature demonstrated that most of the cases have been demonstrated in adults patients with age range from 24 to 78 years. There is a marked preference for female patients. In 2 cases, thyroid paraganglioma was related with synchronous carotid body paraganglioma. Another case showed, the tumor was coexistent with papillary thyroid carcinoma, parathyroid adenoma and bilateral carotid body paraganglioma. Until this point in time, none of the reported cases had been analyzed correctly by radiological examinations before surgery. Ultrasonography ordinarily shows a solid hypoechoic nodule in the thyroid gland with no difference from the rest of the thyroid neoplasms. By thyroid scintiscan, the tumor much of the time shows up as a “cold nodule”. Because of architectural similarity, thyroid paraganglioma might be misdiagnosed as different sorts of thyroid neoplasms. Specifically, the tumor is mostly misdiagnosed as MTC. The two tumors comprise of nesting and organoid cell clusters. The coexpression of neuroendocrine separation makes the distinction further muddled. As the treatment of the disease and follow-up regimen differ greatly, it is fundamental to make a distinction between this two disease patterns. MTC is portrayed by positive staining with calcitonin. The presence of epithelial markers and absence of S100 positive sustentacular cells additionally, facilitate the differential diagnosis [7].

Even though the patient had multiple neck masses, the thyroid hormones were within the normal range, which caused us to think in favor of thyroid paraganglioma. Pre-operative diagnosis in cervical lesions is beneficial in that the surgeon can take precautions against potential hemorrhage and perilesional injury. Non-invasive methods such as ultrasonography, CT Scanning, thyroid scan provide some information. Digital subtraction angiography provides additional information in diagnosing thyroid paraganglioma. In our case, intense enhancement of thyroid mass and abnormal dilated channels behind one of the cervical lymph node raised the suspicion of paraganglioma. Digital subtraction angiogram performed which showed high flow right cervical lesion getting supply from superior thyroidal artery draining into IJV, our working diagnosis was paraganglioma on the basis of these findings.

Fine needle aspiration biopsy of these lesions is a high-risk procedure since the tumor is highly vascular in nature and thus it was not done. In this case, the mass had invaded both the thyroid lobes, and cervical lymph nodes as well as identified during the operation. Two staged procedure was done and the pathological diagnosis was thyroid papillary cancer. Frozen section evaluation of such lesions may be useful. However, histopathology did not explain the enlarged vascular channels in the vicinity of tumor so further immunohistochemistry was done which proves the definitive diagnosis. As the definitive diagnosis in our patient was made after discharge, she was referred to the general surgery clinic for further treatment planning. To this end, the approach to cervical pathologies should be multidisciplinary [8].

Ferri et al., described a case report of primary paraganglioma of thyroid, thyroid ultrasound and fine needle aspiration biopsy were not diagnostic. Preoperative findings were also not confirmatory. Pathological analysis revealed tumor cells positive for chromogranin A, synaptophysin and for S-100. A diagnosis of paraganglioma was finally made. This case highlights the need for immunohistochemistry in the diagnosis of neck masses and to differentiate between intrathyroidal neoplasms and paraganglioma [9]. Furthermore Calo et al., described another difficult case of thyroid paraganglioma associated with chronic lymphocytic thyroiditis and a papillary micro carcinoma. In the following case pre-operative investigations were inconclusive, with even the gross findings insufficient to reach a diagnosis. Histopathology and further IHC confirmed the diagnosis of this elusive condition [10].

## 4 Conclusion

It is mandatory to diagnose the neck masses accurately as treatment modalities of both thyroid papillary carcinoma and paraganglioma is different and so the prognosis also varies. It is well known that papillary thyroid carcinoma has a generally indolent course and prognosis is good if no high risk factors or metastasis are present but differential diagnosis of neck masses should include other lesions also as described in one of the neglected case of thyroid papillary carcinoma which presented with complications and distant metastasis after 7 years of initial diagnosis [11].

In conclusion neck masses should investigated thoroughly by keeping all the differentials in view and in certain specific cases immunohistochemistry is main stay in diagnosing the lesion.

## Funding

There are none, this was a self-funded project.

## Ethical approval

We got ethical approval from our IRB Board.

## Consent

We got consent from the patient’s guardian.

## Author contribution

I am the sole author and only contributor.

## Registration of research studies

N/A.

## Guarantor

Dr. Anchalee Churujana, SiriRaj International Hospital Thailand.

## Provenance and peer review

Not commissioned, externally peer-reviewed.

## Declaration of Competing Interest

There are none.
